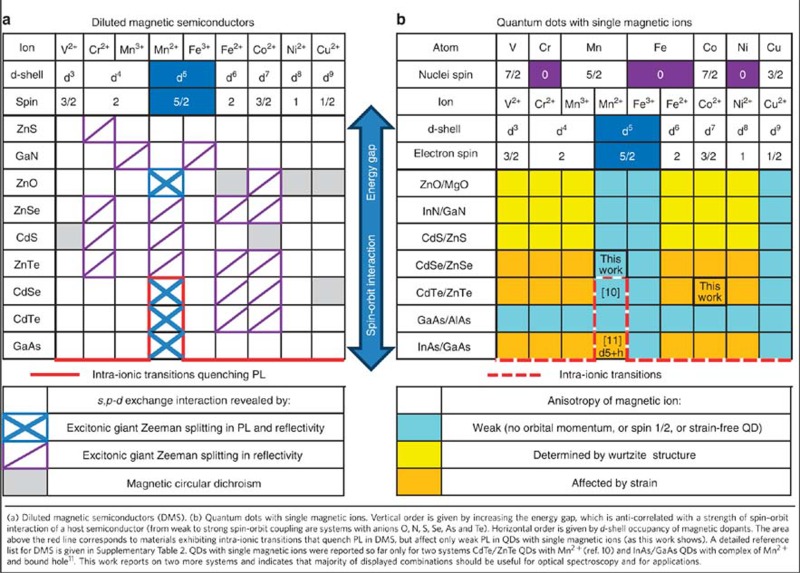# Designing quantum dots for solotronics

**DOI:** 10.1038/ncomms4191

**Published:** 2014-01-27

**Authors:** J. Kobak, T. Smoleński, M. Goryca, M. Papaj, K. Gietka, A. Bogucki, M. Koperski, J.-G. Rousset, J. Suffczyński, E. Janik, M. Nawrocki, A. Golnik, P. Kossacki, W. Pacuski

**Affiliations:** 1 Institute of Experimental Physics, Faculty of Physics, University of Warsaw, Hoża 69, Warsaw 00-681, Poland; 2 These authors contributed equally to this work

## Abstract

Solotronics, optoelectronics based on solitary dopants, is an emerging field of research and technology reaching the ultimate limit of miniaturization. It aims at exploiting quantum properties of individual ions or defects embedded in a semiconductor matrix. It has already been shown that optical control of a magnetic ion spin is feasible using the carriers confined in a quantum dot. However, a serious obstacle was the quenching of the exciton luminescence by magnetic impurities. Here we show, by photoluminescence studies on thus-far-unexplored individual CdTe dots with a single cobalt ion and CdSe dots with a single manganese ion, that even if energetically allowed, nonradiative exciton recombination through single-magnetic-ion intra-ionic transitions is negligible in such zero-dimensional structures. This opens solotronics for a wide range of as yet unconsidered systems. On the basis of results of our single-spin relaxation experiments and on the material trends, we identify optimal magnetic-ion quantum dot systems for implementation of a single-ion-based spin memory.

## Abstract (web summary)

Single-atom dopants embedded in a semiconductor matrix are of potential use for optical, spintronics as well as information storage applications. Here, Kobak *et al.* realize CdTe and CdSe quantum dots with single cobalt and manganese ions and show how the quantum dot design influences single-spin relaxation time.

## Graphical abstract

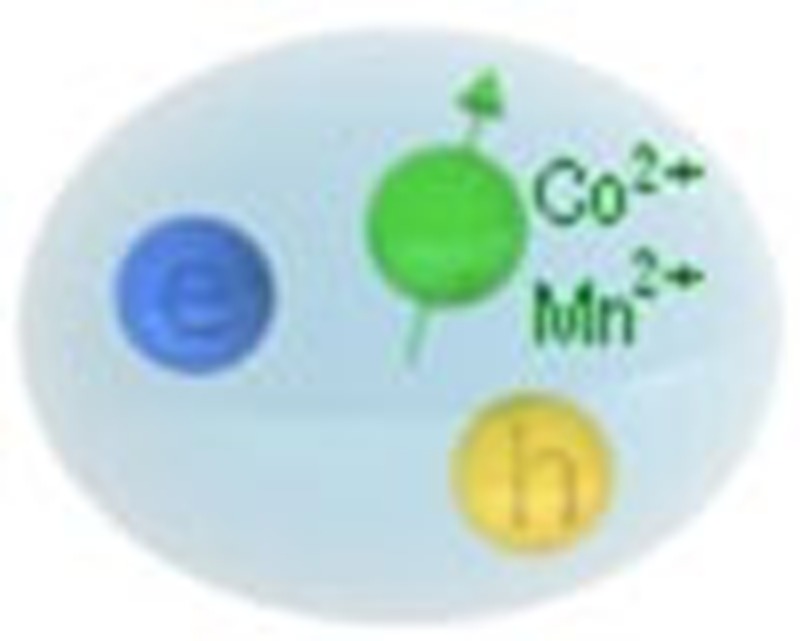

The term solotronics^1^ has been introduced to describe recent advances^2^,^3^ in fabricating and operating semiconductor optoelectronic devices based on single dopants or defects for applications in computer memories, quantum computation and on-demand photon sources. The most advanced solotronics technology has been developed for nitrogen-vacancy (N-V) defect centres in diamond, for which it has been shown that quantum states can be prepared and read out and spin can be manipulated using microwave and optical transitions^4^,^5^,^6^,^7^. Defects similar to N-V centres have also been observed in semiconductors^8^ such as SiC^9^. SiC is more compatible with present semiconductor-based technology than diamond; however, owing to the weak coupling of free carriers to defect centres, it does not allow for electrically controlled operation. More promising in this view is another solotronic system: a single magnetic ion embedded in a semiconductor quantum dot (QD)^10^,^11^,^12^. Here, the spin state of the single ion can be prepared and manipulated both electrically^13^,^14^ and optically^12^,^15^,^16^ through injection of spin-polarized carriers. The *s*,*p*-*d* exchange coupling between the magnetic ion and the band carrier enables an unambiguous readout of the spin projection of the ion from the energy and polarization of a photon emitted by the QD^10^,^11^. The ease of optical addressing of individual QDs enables operation on the level of single ions^12^,^15^. Multiple magnetic ions can be coupled by carriers in one QD^17^,^18^,^19^ or by QDs coupling through tunnelling carriers^12^ or photonic structures^20^. The use of semiconductor heterostructures opens a huge area for testing new ideas for single-ion spin operation, as it offers a band gap and strain engineering, tuning energies of optical and microwave transitions, Fermi level manipulation and integration with p-i-n structures.

A severe limitation of QDs doped with transition metal ions was attributed to the efficient recombination channel introduced by magnetic ions^21^,^22^,^23^,^24^,^25^,^26^,^27^,^28^,^29^ when the exciton energy is higher than the intra-ionic transition energy, which should result in quenching of exciton emission. Therefore, the only QD systems with single-magnetic ions considered so far were those where the intra-ionic transition energies exceed the exciton energy, namely Mn^2+^ embedded in CdTe/ZnTe and InAs/GaAs QDs^10^,^11^,^12^,^13^,^14^,^15^,^16^,^30^,^31^,^32^. On the other hand, incorporation of magnetic ions such as Cr, Fe, Co, Ni or Cu would bring physical properties like orbital momentum, reduced number of spin states, sensitivity to local strain, the Jahn-Teller effect or isotopes with zero nuclear spin, offering additional degrees of freedom for designing quantum states. Extending the studies of single magnetic ions to other QD systems such as CdSe, ZnSe, CdS, ZnS, ZnO, GaN or other wide-gap semiconductors would offer, in turn, increased photoluminescence (PL) efficiency at higher temperatures, enhancement of the exchange interaction within excitons and between excitons and ions or reduction of spin-orbit coupling and the resulting spin relaxation rates.

We report here for the first time on a single cobalt ion in a CdTe/ZnTe QD and a single manganese ion in a CdSe/ZnSe QD. The spin states of the dopant ions are mapped onto the QD optical transitions recorded in a magneto-PL measurement. We employ PL decay measurements to demonstrate that contrary to the case of systems with many magnetic ions^21^,^22^,^23^,^24^,^25^,^26^,^27^, the exciton emission quenching is negligible for single dopants. Through modulated, polarization-resolved PL measurements we access the single-spin relaxation and prove that all-optical control of a single magnetic moment is feasible in the systems studied. Moreover, we show that spin properties of magnetic QDs can be designed by an independent choice of the magnetic ion and the QD material. We discuss the role of the electronic configuration of *d*-shell, spin-orbit and hyperfine interactions and, finally, we indicate the most promising design of future QD-based solotronic systems.

## Results

### PL spectra of QDs with single magnetic ions

**Figure 1:**
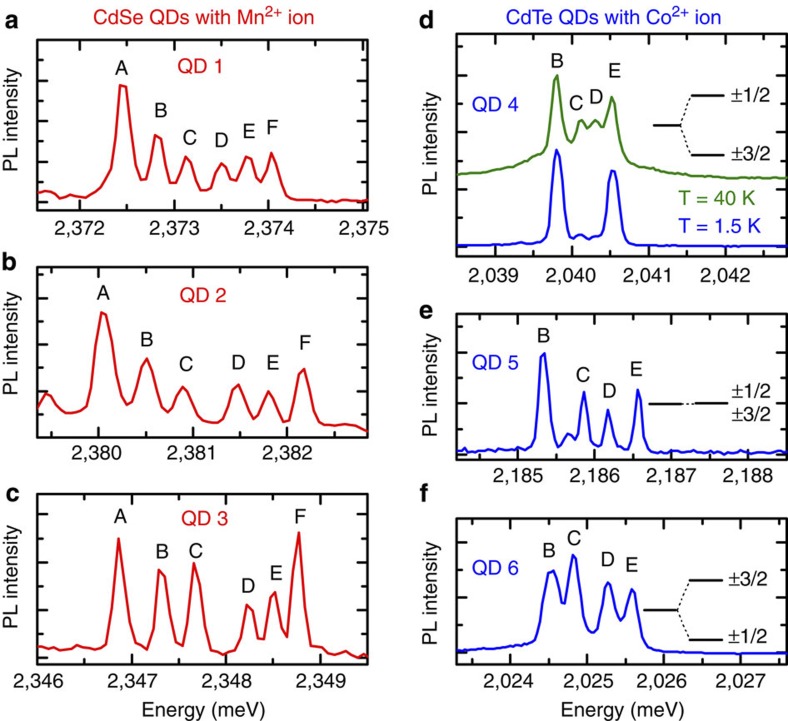
Excitonic PL spectra of quantum dots with single magnetic ions. (**a**–**c**) PL spectra of CdSe QDs with single Mn^2+^ ions. (**d**–**f**) PL spectra of CdTe QDs with single Co^2+^ ions. Spectra are measured at zero magnetic field and low temperature, *T*=1.5 K. The exciton splitting results from the *s*,*p*–*d* exchange interaction with a magnetic ion. For QD with Mn^2+^ ion (*S*=5/2), we observe six components that in σ^±^ circular polarization correspond to Mn^2+^ spin projections ∓5/2 (A), ∓3/2 (B), ∓1/2 (C), ±1/2 (D), ±3/2 (E) and ±5/2 (F) while for QD with Co^2+^ ion (*S*=3/2), there are four components related to Co^2+^ spin projections ∓3/2 (B), ∓1/2 (C), ±1/2 (D) and ±3/2 (E). Intensities of various lines are related to a strain and resulting occupancy of magnetic ion states. For Mn^2+^, the effect of the strain is negligible (**a**–**c**) while for Co^2+^ the impact is visible (**d**–**f**), for example, cobalt shown in (**d**) has a fundamental state with spin ±3/2, so observation of ±1/2 states requires an increase in temperature. The upper curve in (**d**) was blueshifted by 4.42 meV in order to compensate for the temperature shift.

Samples with self-assembled QDs containing magnetic ions are grown by molecular beam epitaxy (see Methods) and studied by PL at low temperatures (down to 1.5 K). The use of a microscope objective enables the observation of exciton emission lines of individual QDs. Among sharp lines with a typical emission pattern of nonmagnetic QDs^33^ (majority) and broader lines related to QDs with many magnetic ions^19^,^22^,^34^, it is possible to identify the emission multiplets characteristic of individual QDs with exactly one magnetic ion, where the ion spin state is probed by confined excitonic complexes (neutral and charged excitons, biexciton and so on). In particular, the emission related to a bright state of a neutral exciton (Fig. 1) consists of a set of lines split by the *s*,*p*–*d* exchange interaction. The number of lines is determined by the possible magnetic ion spin projections on the growth axis, this being the exciton quantization axis. More specifically, for Mn^2+^ with spin 5/2 there are six spin projections: ±5/2, ±3/2 and ±1/2, so we observe six lines for a CdSe QD with a single Mn^2+^ ion (Fig. 1a,b,c), analogously to a CdTe QD with the same ion^10^. The Co^2+^ ion spin is 3/2 and thus there are four spin projections: ±3/2 and ±1/2, resulting in four lines for a CdTe QD with a single Co^2+^ ion (Fig. 1d–f). However, the intensity of the lines related to Co^2+^ spin projections ±3/2 (outer lines) can be significantly different from those related to the spin projections ±1/2 (inner lines). The Co^2+^ ion orbital momentum is non-zero, and Co^2+^ incorporated in the crystal is very sensitive to local anisotropy and strain, which lead to the splitting of ±3/2 and ±1/2 states and a difference in their occupancy^35^,^36^,^37^. The QD shown in Fig. 1d exhibits outer lines more intense than the inner lines. This means that in this case the strain makes the state with spin ±3/2 the ground state. The difference between the spin state occupancies (and therefore the exciton line intensities) is more pronounced at low temperatures, as expected from Boltzmann statistics.

**Figure 2:**
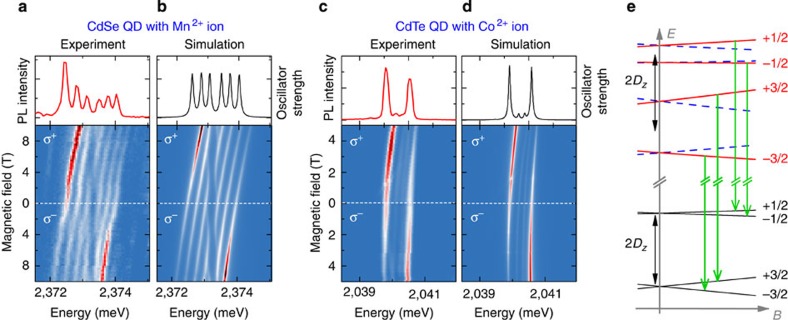
Magneto-optical spectroscopy of QDs with single magnetic ions. *T*=1.5 K. PL of an exciton in a CdSe QD with a Mn^2+^ ion as a function of magnetic field in Faraday configuration (**a**) and a corresponding simulation (**b**). An analogous experiment (**c**) and simulation (**d**) for a CdTe QD with Co^2+^. More intense low-energy lines in the polarization σ^+^ and high-energy lines in the polarization σ^−^ at high a magnetic field indicate alignment of ion spins along the external magnetic field direction. Parameters used for calculation of (**b**) and (**d**) are listed in Supplementary Table 1. The scheme of excitonic transitions (**e**) in σ^+^ polarization is shown for a QD with Co^2+^ for a relatively simple case, where the strain-induced Co^2+^ anisotropy axis is parallel to the growth axis and the resulting zero-field spin splitting of Co^2+^ states is equal to 2*D*_*z*_, which is in the microwave range, of the order of 1 meV. The spin projection of Co^2+^ is indicated.

The identification of the excitonic lines is confirmed by the analysis of PL spectra measured as a function of the magnetic field (Fig. 2). Zeeman shifts of bright and dark excitonic transitions for a CdSe QD with Mn^2+^ (Fig. 2a) can be well described (Fig. 2b) using the model proposed for a CdTe QD with Mn^2+^ (ref. 10). In order to account for all the observed features of the PL spectrum from a CdTe QD with a single Co^2+^ (Fig. 2c,d), we extend the model by introducing a strain vector that induces a zero-field splitting of the Co^2+^ spin states (see Methods). Figure 2e shows the scheme of excitonic optical transitions for a relatively simple case, when the strain-induced Co^2+^ anisotropy axis is parallel to the growth axis. In plane anisotropy of Co^2+^ would induce an additional excitonic mixing, analogous to the case of a neutral Mn centre (*d*^5^+*h*) in a InAs/GaAs QD^31^. Typical PL spectra of various QDs with single-magnetic ions are presented in Supplementary Note 1 and Supplementary Figs 1 and 2.

### Recombination channels for excitons

Quenching of exciton PL by a recombination channel introduced by a magnetic dopant is an important obstacle in studying diluted magnetic semiconductors (DMS) with an energy gap larger than the magnetic ions’ internal transition energies^25^,^26^,^28^. This is the case for DMS with magnetic ions other than Mn (for example, V, Cr, Fe, Co, Ni and Cu)^36^,^38^,^39^. Only Mn^2+^ ions have relatively large intra-ionic transition energies (about 2.2 eV) allowing for efficient PL studies of excitons in Cd_1−*x*_Mn_*x*_Te^40^,^41^ (energy gap *E*_g_(CdTe)=1.6 eV) and Ga_1−*x*_Mn_*x*_As with low Mn concentration (*x*
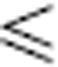
0.13%)^42^,^43^ (energy gap *E*_g_(GaAs)=1.5 eV). As a consequence, the first approaches to studies of dots with single magnetic ions were limited to manganese and QDs with low emission energy: CdTe/ZnTe^10^ and InAs/GaAs^11^. The relatively small energy gap of CdSe (*E*_g_(CdSe)=1.7 eV) results in energy transfer from the magnetic ions to excitons^24^ and enables PL studies of bulk Cd_1−*x*_Mn_*x*_Se^44^. In the case of nanostructures, quantum confinement increases the exciton energy. As a consequence, the direction of the energy transfer is reversed, leading to PL quenching^22^,^23^,^24^,^25^,^27^ and a significant shortening of the exciton lifetime^21^,^28^.

**Figure 3:**
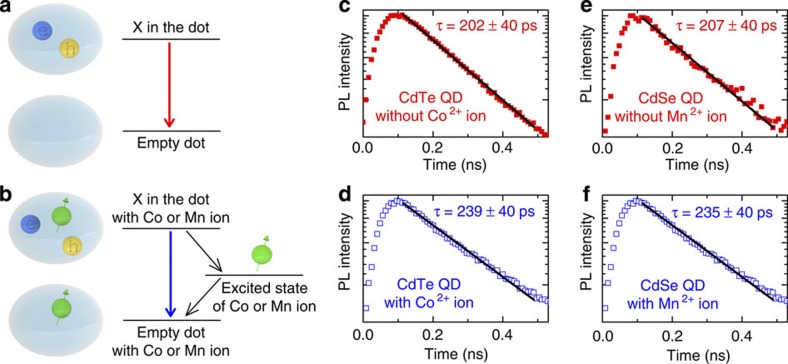
Exciton recombination channels in a QD with a single magnetic ion. (**a**) Radiative channel in a nonmagnetic QD, (**b**) radiative and nonradiative channels in a QD with a magnetic ion, which exhibits intra-ionic transitions at energies lower than the exciton energy. Exciton PL decay measurement for a CdTe QD (**c**), CdTe QD with a single Co^2+^ (**d**), CdSe QD (**e**) and CdSe QD with a single Mn^2+^ (**f**). We do not observe any impact of single dopants on exciton decay time (*τ*), which implies that the nonradiative channel is slower than the radiative channel. Temporal resolution of the set-up is 40 ps. Data are collected at zero magnetic field and low temperature, *T*=1.6 K.

However, our measurements of the PL decay performed on the CdTe/ZnTe sample reveal the same lifetime of about 220±40 ps for a QD with and without a single Co^2+^ ion (see Fig. 3c,d). Also, for selenide QDs exciton lifetime is measured to be around 220±40 ps for a dot with and without a single Mn^2+^ (Fig. 3e,f). Therefore, we do not observe shortening of the exciton lifetime induced by single magnetic ions. Moreover, the PL intensity of individual QDs with single magnetic ions is comparable to that of nonmagnetic QDs. The above findings indicate that the quenching of excitonic emission is not efficient when single dopants are introduced to QDs. We interpret this as the result of the discrete density of states of zero-dimensional systems with exactly one magnetic ion. In this case the exciton energy cannot be efficiently transferred either to phonons or to the magnetic ion owing to an energy mismatch. This effect can be compared with the phonon bottleneck that can hinder exciton relaxation to the ground state of zero-dimensional systems^45^,^46^ if other relaxation channels, such as the Auger process^47^,^48^,^49^, are not efficient. Furthermore, energy transfer from exciton to magnetic ions can also be hindered by spin conservation selection rules^50^,^51^,^52^.

The high efficiency of radiative exciton recombination found in the present work is different with respect to the bulk DMS case, where the exciton is typically coupled to an ensemble of magnetic ions, able to absorb energy over a wide range, for example, by a collective change of spin configuration. The above results not only show that PL studies of DMS can be significantly extended by using zero-dimensional structures, but they also imply high-fidelity optical readout of a single-dopant quantum state in a QD.

### Spin relaxation of a single magnetic ion

**Figure 4:**
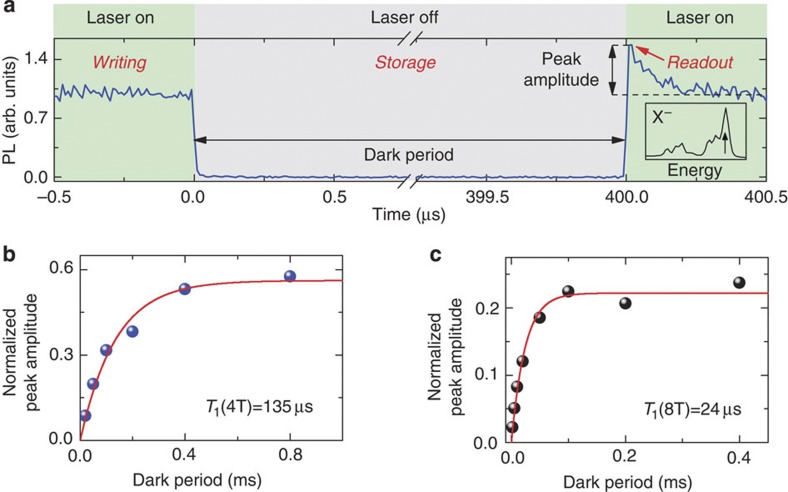
Relaxation dynamics of a single Mn^2+^ ion spin in a CdSe/ZnSe QD. *T*=1.6 K. (**a**) Temporal profile of the PL intensity of the highest energy X^−^ emission line (indicated in the inset) in magnetic field *B*=4 T (detection in σ^−^ polarization). The nonresonant (405 nm) continuous-wave excitation is subsequently turned on and off for controlled periods of time. The amplitude of the PL intensity peak observed immediately after turning the excitation on indicates the loss of information stored on the Mn^2+^ spin. (**b**,**c**) The amplitude of the PL intensity peak measured just after turning on the excitation versus length of the dark period for external magnetic fields of 4 T and 8 T, respectively. The fitted exponential curves yield the storage times *T*_1_ of information on the Mn^2+^ ion spin.

In order to investigate the spin relaxation dynamics of a Mn^2+^ ion embedded in a CdSe/ZnSe QD, we measure time-resolved PL in a magnetic field, with an on-/off-modulated non-resonant laser excitation (Fig. 4). When the laser is switched on, the optically created excitons injected into the QD depolarize the spin of the embedded magnetic ion (typically within several hundreds of nanoseconds). Then the laser is switched off for a dark period, during which the Mn^2+^ spin approaches the state of alignment to the magnetic field direction. Finally, the laser is switched on again to perform the readout of the spin state by measuring the temporal increase of the PL amplitude of a high-energy line. This line corresponds to the state of the Mn^2+^ ion with spin oriented parallel to the magnetic field. Figure 4b,c shows the dependence of the PL amplitude on the length of the dark period for two different values of magnetic field. The exponential fits allow us to determine the Mn^2+^ ion spin relaxation times in a CdSe QD at helium temperature, in magnetic fields of 4 T and 8 T to be equal to 135 μs and 24 μs, respectively. It is over an order of magnitude longer than the relaxation time of the Mn^2+^ ion in a CdTe QD, that is 5 μs for *B*=4 T measured by Goryca *et al.* (private communication, 2013) with the technique reported in ref. 12. Such results demonstrate that modification of the QD material can significantly increase the storage time of information written on the Mn^2+^ spin, which can noticeably improve the features of quantum memories based on QDs with single magnetic ions. On the other hand, embedding an ion with a different electronic configuration may lead to an acceleration of the spin dynamics. In particular, similar measurements to those described in Fig. 4 performed on a CdTe QD with a single Co^2+^ ion yield a relaxation time of about 2 μs in a magnetic field of 3 T, shorter than for a Mn^2+^ ion in a CdTe QD.

The method of single-spin relaxation measurement based on initial depolarization of magnetic ions presented here has the advantage of being applicable to any QD system with magnetic ions, independently of the choice of photoexcitation energy. However, for particular systems, measurements can be improved by the identification of resonant excitation channels, which allow for efficient transfer of polarization to QDs, and consequently optical polarization of the magnetic ions. Such resonant excitation channels have been identified for (Ga,Mn)As-based quantum wells^43^, for CdTe QDs with single Mn ions^12^,^15^,^53^ and for InAs/GaAs QDs with a single Mn^16^. For CdSe/ZnSe QDs even quasi resonant excitation results in some degree of QD exciton polarization^54^. This effect and identification of resonant excitation channels for a CdSe QD with a single Mn will result in efficient optical orientation of Mn and will be an important step towards observation of coherent phenomena.

## Discussion

The results presented above highlight the opportunity for extension of QD-based solotronics research to as yet unexplored combinations of semiconductor systems and magnetic ions. In this section, we discuss the optimal systems from the point of view of practical implementation of QD-based solotronic devices. Materials where ion–carrier exchange interaction has been experimentally observed are summarized in Table 1a. Table 1b presents the most interesting combinations of QDs and magnetic ions. So far, only two systems have been studied (single Mn in CdTe/ZnTe and InAs/GaAs QDs). With our work we introduce a new magnetic ion—Co^2+^ and a new solotronic system—CdSe/ZnSe.

We note first that for applications in solotronics it is important to lenghten the single-spin relaxation time using magnetic ions without orbital momentum, that is, with a half-filled *d*-shell (*d*^5^). At non-zero magnetic fields, the Mn^2+^ ions (*d*^5^) in a bulk matrix exhibit a relaxation time longer by two orders of magnitude than for other 3*d* transition metals^55^. This trend is confirmed in our work for the ions in QDs, since Co^2+^ (*d*^7^ shell) exhibits a shorter relaxation time than Mn^2+^. However, since increasing magnetic field enhances spin relaxation^55^ (see Fig. 4), practical applications of solotronic devices will be realized at zero or low magnetic fields. At zero field, the nuclear spin^12^,^15^,^32^,^41^,^56^ plays the most important role and we predict that another *d*^5^ ion, Fe^3+^ with zero nuclear spin, should be more stable than Mn^2+^ with 5/2 nuclear spin. With Fe^3+^ and a QD built from isotopically purified group II and VI elements (which are attainable), it will be possible to have a whole system free of nuclear spin and to obtain an extremely long spin relaxation time, as was shown for N-V centres in isotopically engineered diamond^5^.

The rate of spin relaxation of isolated ions increases with the strength of the spin–orbit interaction^40^,^55^,^56^, which depends on the magnetic ion and the host material^57^. Therefore it is reasonable to use QDs based on semiconductors with light anions: sulphides, oxides and nitrides, as they exhibit a weak spin–orbit interaction. This trend^58^ is confirmed by our results on QDs, as single Mn^2+^ has a longer relaxation time when it is embedded in a selenide (Fig. 4) than in a telluride^12^ (heavier) (Goryca *et al.* private communication, 2013) system. For ensembles of isolated Fe^3+^ and Mn^2+^ in oxides and sulphides, long spin relaxation times up to 0.4 s^59^,^60^ and a spin coherence time of 0.9 μs^61^ were demonstrated at helium temperatures.

Manipulation of the stored spin is equally important for application as a long spin relaxation time. Manipulation through the ion–carrier exchange interaction^12^ or microwave radiation^61^ should be feasible for all the systems presented in Table 1b. Another, thus-far-unexploited possibility for single-magnetic-ion spin manipulation in QDs, is related to intra-ionic transitions, which are similar to transitions exploited for defect centres^8^. The intra-ionic transitions could be useful for reading, manipulation and writing of the magnetic ion spin. Spin readout from sharp intra-ionic transitions of Co^2+^ ions in Zn_1−*x*_Co_*x*_O layers has been demonstrated in ref. 37.

It is worth noting that strain gives the possibility of inducing a temporal evolution of spin, tuning the zero-field splitting and reducing the spin degeneracy of the ground state. Thus, it can be profitable for manipulation of ion spins. The effect of strain on Co^2+^ spin states is shown in Fig. 2e. In order to use strain or crystal field effects, it is desirable to control the anisotropy axis, for example, by using wurtzite structure compounds, where the *c* axis is expected to define the quantization axis of the zero-field spin splitting. If one wishes, in turn, to eliminate a strain-induced complexity, lattice-matched materials, such as GaAs and AlAs for growth of magnetic QDs by droplet epitaxy, are recommended.

In conclusion, the optical properties of a single Co^2+^ ion in a CdTe QD and a single Mn^2+^ ion in a CdSe QD are presented for the first time. The QD emission decay time is found to be equal in the case of magnetic ion doped and undoped QDs. This indicates that quenching of the QD emission is negligible in zero-dimensional systems with single magnetic ions. A single manganese in a CdSe QD exhibits the longest spin relaxation time among the single-magnetic-ion-QD systems optically investigated so far. As such, it is clearly a good candidate for a single-spin memory. However, the most promising systems for single-spin memories have still not been tested experimentally: systems with a weak spin–orbit interaction, a wide energy gap and with *d*^5^ magnetic ions, that is, self-assembled QDs based on oxides, sulphides or nitrides with a single Mn^2+^ or Fe^3+^. Further experimental and theoretical studies are needed in order to indicate the optimal QD system for manipulation of a single-magnetic-ion spin.

## Methods

### Growth of samples with self-assembled QDs containing single magnetic ions

Samples are grown on GaAs (100)-oriented substrates using molecular beam epitaxy. After about a 1-μm thick buffer layer of ZnTe (or ZnSe), about three monolayers of CdTe (or CdSe) were grown using atomic layer epitaxy by alternate cycles of Cd and Te (or Se). In the middle of the CdTe (or CdSe) thin layer, a very small (previously calibrated by giant Zeeman splitting measurements of layers such as Zn_0.997_Co_0.003_Te) amount of magnetic ions: Co or Mn are introduced during 3 s of deposition. Next, the sample is cooled down for 1 h in Te (or Se) molecular flux, so at the end sample is covered with amorphous Te (or Se), which helps the QDs to form^34^,^62^. Subsequently the sample is heated up to the growth temperature, to sublimate the amorphous Te (or Se). The QDs are covered with a 100 nm ZnTe (or ZnSe) cap. No mesas or masks are needed to limit the number of observed QDs. The emission from single QDs at temperature of 2 K is collected with a microscope objective assuring a resolution of 0.5 μm.

### Modelling of magneto-PL

A quantitative description of measured QDs magneto-PL spectra (Fig. 2) is provided by a model of the neutral exciton inside a QD with a single magnetic dopant in magnetic field (*B*) parallel to the growth axis (*z*). We consider initial and final states of PL transition (Fig. 2e). For the initial state, the model takes into account the energy of an exciton in a nonmagnetic QD (isotropic and anisotropic electron–hole exchange interactions, heavy-light hole mixing, Zeeman effect and diamagnetic shift), energy of ion–electron and ion–hole exchange interactions (*s,p–d* interactions), and finally the magnetic ion energy determined by the Zeeman effect and the strain vector. For the final state, we consider only the magnetic ion energy.

The above model is described by the following Hamiltonian *H*_X,M_(*B*):


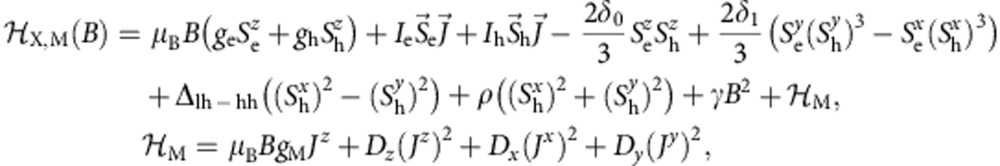


where *H*_M_ is the Hamiltonian of the magnetic ion, *g*_e_, *g*_h_ and *g*_M_ are electron, hole and magnetic ion *g* factors, 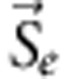, 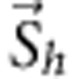 and 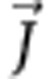 are electron, hole and magnetic ion spin operators, *I*_e_ and *I*_h_ are the electron-ion and hole-ion exchange constants^10^,^63^, *δ*_0_ and *δ*_1_ are the energies related to isotropic and anisotropic parts of the electron–hole exchange interaction^64^, Δ_lh−hh_ is the heavy-light hole splitting, *ρ* represents the strength of the valence-band mixing^65^,^66^ and *γ* is an excitonic diamagnetic shift constant. The parameters *D*_*x*_, *D*_*y*_, *D*_*z*_ describe strain-induced zero-field splitting and the anisotropy axis of Co^2+^ ion spin states. In the case of Mn^2+^ ion, strain effects^67^ are much weaker and zero-field splitting is neglected. Supplementary Table 1 lists example parameters of the Hamiltonian. These parameters were used for calculation of spectra of CdSe QD with single Mn^2+^ and CdTe QD with single Co^2+^ presented in Fig. 2b,d. Parameter *g*_M_ for a given ion is from literature data^68^,^69^. Other parameters were determined from a fit to the data presented in Fig. 2a,c. The oscillator strength of each optical transition is calculated for initial state given by *H*_X,M_ and final state given by *H*_M_. Line intensity is calculated as a multiplication of oscillator strength and magnetic ion spin state occupancy given by the Boltzmann distribution for an effective temperature *T*_eff_. In this simple model, relaxation of the exciton–ion complex is neglected.

## Author contributions

J.K., M.P., K.G., J.-G.R., E.J. and W.P. grew and characterized samples. J.K., T.S., M.K., M.G., A.G., P.K. and W.P. performed magneto-optical experiments, data analysis and modelling. T.S., A.B. and M.G. performed single-spin relaxation measurements. T.S., J.K., J.S., M.N., A.G. and W.P. prepared the manuscript in consultation with all authors.

## Additional information

**How to cite this article:** Kobak, J. *et al.* Designing quantum dots for solotronics. *Nat. Commun.* 5:3191 doi: 10.1038/ncomms4191 (2014).

## Supplementary Material

Supplementary Information Supplementary Figures 1-2, Supplementary Tables 1-2, Supplementary Note 1 and Supplementary References

## Figures and Tables

**Table 1:** Tables summarizing semiconductor systems where interaction of excitons and magnetic ion has been confirmed using optical spectroscopy.