# Supplementary material for: Designing quantum dots for solotronics
Source: Nat Commun. 2014 Jan 27;5:3191. doi: 10.1038/ncomms4191 (PMC3916836; doi:10.1038/ncomms4191)
Supplement: Supplementary Information — Supplementary Figures 1-2, Supplementary Tables 1-2, Supplementary Note 1 and Supplementary References [file ncomms4191-s1.pdf]

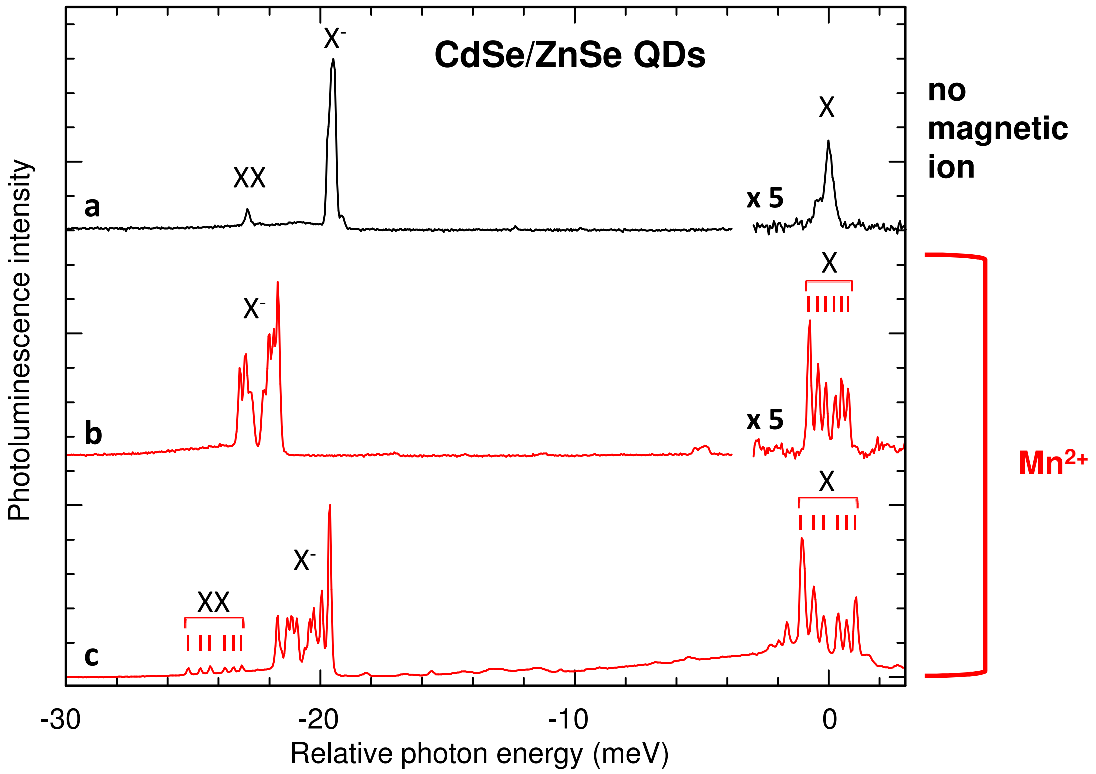

Supplementary Fig. 1: PL spectra of individual CdSe/ZnTe QDs. $T = 1.5$ K. Lines related to neutral exciton (X), charged exciton (X^-) and biexciton (XX) are marked. (**a**) QD without magnetic ion. (**b,c**) QDs with single Mn^{2+}. Principal six lines of biexciton and neutral bright exciton originates from *s*,*p*-*d* exchange interaction of exciton with Mn^{2+} ion. Additional weak lines below X in (**c**) are related to dark-exciton states. Energies of the neutral exciton emission are equal to 2318 meV (**a**), 2373 meV (**b**), and 2381 meV (**c**).

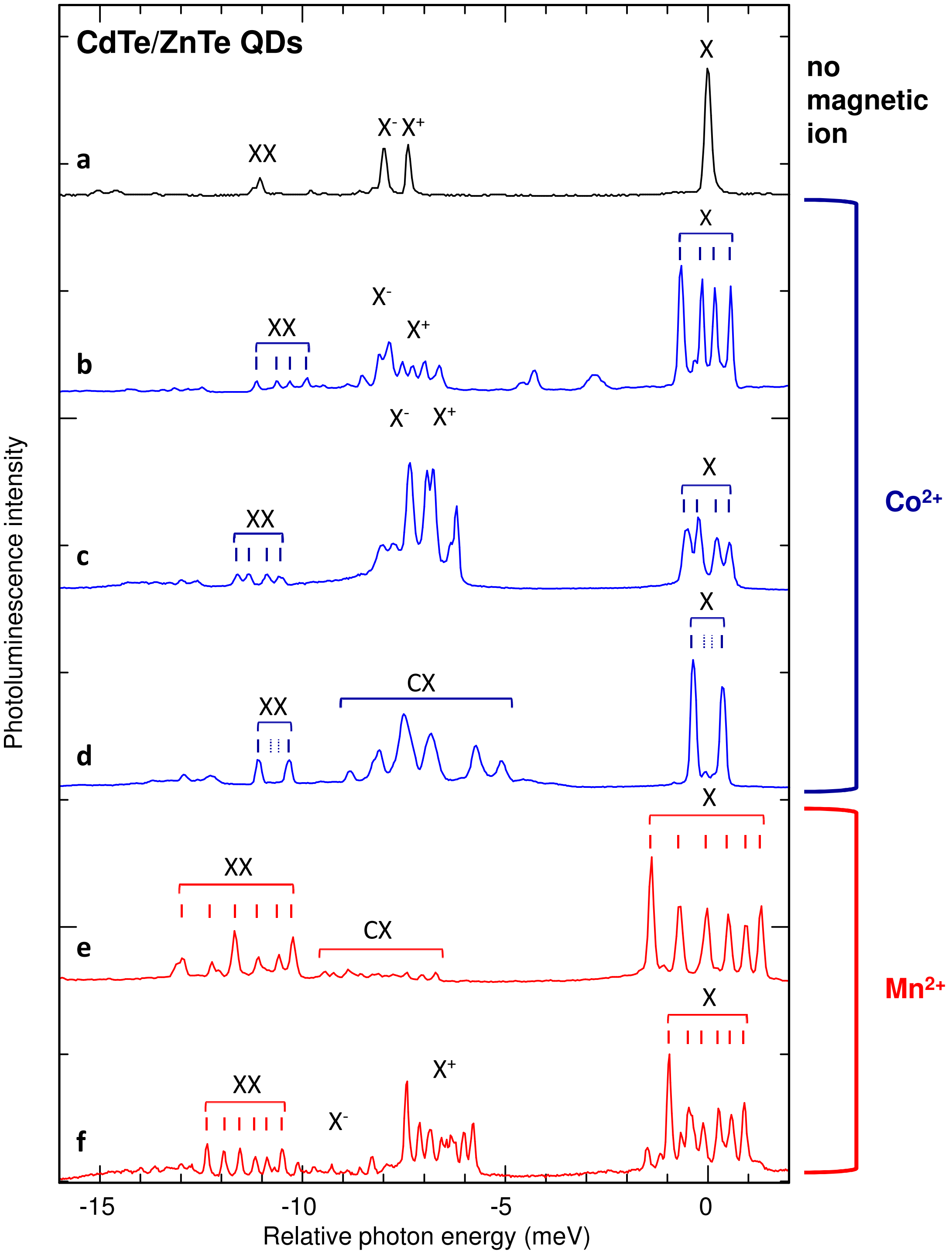

Supplementary Fig. 2: PL spectra of individual CdTe/ZnTe QDs. $T = 1.5$ K. Lines related to neutral exciton (X), charged excitons (X^- and X^+), overlapping charged excitons (CX) and biexciton (XX) are marked. (**a**) QD without magnetic ion. (**b,c,d**) QDs with single Co^{2+}. (**e,f**) QDs with single Mn^{2+}. Due to exchange interaction between exciton and magnetic ion neutral exciton and biexciton states split and we observe four related emission lines in case of QDs with a single Co^{2+} (**b,c,d**) or six emission lines in case of QDs with a single Mn^{2+} (**e,f**). Additional weak lines below X in (**f**) are related to a strong mixing of dark-exciton and bright-exciton states. In spectra (**b,c,d**), intensity of various components of exciton in CdTe QD with Co^{2+} depends on strain induced zero-field splitting of Co^{2+} spin states $\pm 1/2$ and $\pm 3/2$. Energies of the neutral exciton emission are equal to 2185 meV (**a**), 2186 meV (**b**), 2025 meV (**c**), 2040 meV (**d**), 2214 meV (**e**), and 1961 meV (**f**).

Ion	QD	I_e meV	I_h meV	δ_0 meV	δ_1 meV	$\frac{\rho}{\Delta_{lh-hh}}$	γ μeV/T^2	g_e	g_h	g_M	D_x meV	D_y meV	D_z meV	T_{eff} K
Mn^{2+}	CdSe	-0.015	0.195	1.0	0.23	0	0.5	-0.4	0.35	2.0	0	0	0	30
Co^{2+}	CdTe	-0.06	0.16	0.8	0.01	0.5	3.2	-0.35	0.2	2.3	0	-0.3	-1.4	10

Supplementary Table 1: Parameters of the model used in calculation of QDs spectra presented in Figs. 2b,d of the main text. Parameters listed in the table can be compared to parameters already reported and discussed in literature devoted to CdTe/ZnTe QDs with single Mn[10,30,70,71], nonmagnetic QDs CdTe/ZnTe[33,72–74], and nonmagnetic QDs CdSe/ZnSe[75,76]. New in this work is determination of parameter D for single cobalt in QD. We note that for QD discussed in this work, which is rather typical CdTe QD with Co^{2+}, $D_z = -1.4$ meV, so it is negative, and its absolute value is larger than D values observed in wurtzite structure DMSs with Co^{2+}: 0.062 meV for CdSe[77], 0.084 meV for CdS[77], and 0.34 meV for ZnO[37,78,79].

Ion	V^{2+}	Cr^{2+}	Mn^{3+}	Mn^{2+}	Fe^{3+}	Fe^{2+}	Co^{2+}	Ni^{2+}	Cu^{2+}
d-shell	d^3	d^4	d^4	d^5	d^5	d^6	d^7	d^8	d^9
ZnO				80,81		82	37	83*	83*
ZnS		84							
ZnSe		84		85		86	87		
ZnTe		84		88		89	90		
CdS	91	92		93			94*		
CdSe				95		96	97		29*
CdTe				98		99	100,101		
GaN			102,103		104				
GaAs				42,105					

Supplementary Table 2: Summary of references related to the magneto-optical observation of the excitonic giant Zeeman splitting in bulk diluted magnetic semiconductors. Additionally, references related to MCD study of nanocrystals with magnetic ions are shown with a star (*). The table is an updated set of data from Ref. 63. Significant number of studied DMS systems indicates area for engineering QDs with single magnetic ions. Before our work, only Mn^{2+} was used as a single magnetic ions in CdTe QDs[10,12–15,30,32,34,53,70,106–112] and InAs QDs[11,16,31,113]. This work presents two new systems, CdSe QDs with Mn^{2+} and CdTe QDs with Co^{2+}, and indicates that majority of displayed combinations should be useful for optical spectroscopy as well as for practical applications.

Supplementary Note 1: Zero field PL spectra of various QDs with single magnetic ions

In Supplementary Figs. 1 and 2 are presented examples of photoluminescence spectra of individual CdSe/ZnSe and CdTe/ZnTe QDs with single magnetic ions (Mn^{2+} and Co^{2+}) and without magnetic ions. At low excitation power the line of neutral exciton (X) and one or two lines of charged excitons (X^- and X^+) are observed. At higher excitation power, it is usually possible to resolve biexciton line (XX) and lines related to higher excitonic complexes (not marked in Figs. S1 and S2). The order in emission energy (from higher to lower energies) is: the exciton line, the X^+, next the X^-, and finally the XX. Such order is kept for all the observed QDs, regardless of the QD material. Since selenides tend to be n-type materials we do not observe, however, positively charged exciton line for CdSe/ZnSe QDs. Exciton - biexciton separation energy is about 11 meV for CdTe QDs and about 24 meV for CdSe QDs.

Identification of excitonic emission lines for nonmagnetic CdTe and CdSe QDs was extensively discussed in literature[33,73,114–116]. In order to confirm our identification, in addition to the comparison of the relative positions of the excitonic transitions to the literature ones, dependence of the line position on the detected linear polarisation angle, line intensity dependence on the excitation power, and evolution of the excitonic lines in magnetic field was analyzed. QDs with single magnetic ions exhibit lines split by s,p-d exchange interactions. The neutral exciton and biexciton lines typically exhibit 6 components for Mn^{2+} (spin 5/2) and 4 components for Co^{2+} (spin 3/2) as marked in the figures. Intensity of various components of exciton in CdTe QD with Co^{2+} depends on strain and temperature. In some cases mixing with dark-excitons[10,30] leads to observation of more lines for neutral exciton, as shown in the Supplementary Figs. 1c and 2f.

Supplementary References

[70] Besombes, L., Léger, Y., Maingault, L., Ferrand, D., Mariette, H. & Cibert, J. Carrier-induced spin splitting of an individual magnetic atom embedded in a quantum dot. *Phys. Rev. B* **71**, 161307 (2005).

[71] Besombes, L., Léger, Y., Maingault, L., Ferrand, D., Bougerol, C., Mariette, H. & Cibert, J. Optical properties of manganese-doped individual CdTe quantum dots. *Acta Phys. Pol. A* **108**, 527–540 (2005).

[72] Smoleński, T., Kazimierczuk, T., Goryca, M., Jakubczyk, T., Kłopotowski, L., Cywiński, L., Wojnar, P., Golnik, A. & Kossacki, P. In-plane radiative recombination channel of a dark exciton in self-assembled quantum dots. *Phys. Rev. B* **86**, 241305 (2012).

[73] Kazimierczuk, T., Smoleński, T., Kobak, J., Goryca, M., Pacuski, W., Golnik, A., Fronc, K., Kłopotowski, L., Wojnar, P. & Kossacki, P. Optical study of electron-electron exchange interaction in CdTe/ZnTe quantum dots. *Phys. Rev. B* **87**, 195302 (2013).

[74] Hewaparakrama, K. P., Mackowski, S., Jackson, H. E., Smith, L. M., Heiss, W. & Karczewski, G. Tuning spin properties of excitons in single CdTe quantum dots by annealing. *Nanotechnology* **19**, 125706 (2008).

[75] Akimov, I. A., Kavokin, K. V., Hundt, A. & Henneberger, F. Electron-hole exchange interaction in a negatively charged quantum dot. *Phys. Rev. B* **71**, 075326 (2005).

[76] Hundt, A., Flissikowski, T., Lowisch, M., Rabe, M. & Henneberger, F. Excitation spectrum, relaxation and coherence of single self-assembled CdSe quantum dots. *Phys. Stat. Sol. B* **224**, 159–163 (2001).

[77] Lewicki, A., Schindler, A. I., Miotkowski, I., Crooker, B. C. & Furdyna, J. K. Specific heat of $Cd_{1-x}Co_xS$ and $Cd_{1-x}Co_xSe$ at low temperatures. *Phys. Rev. B* **43**, 5713–5718 (1991).

[78] Jedrecy, N., Bardeleben, H. J. V., Zheng, Y. & Cantin, J. L. Electron paramagnetic resonance study of $Zn_{1-x}Co_xO$: A predicted high-temperature ferromagnetic semiconductor. *Phys. Rev. B* **69**, R041308 (2004).

[79] Sati, P., Hayn, R., Kuzian, R., Régnier, S., Schäfer, S., Stepanov, A., Morhain, C., Deparis, C., Laügt, M., Goiran, M. & Golacki, Z. Magnetic anisotropy of Co^{2+} as signature of intrinsic ferromagnetism in ZnO:Co. *Phys. Rev. Lett.* **96**, 017203 (2006).

[80] Przeździecka, E., Kamińska, E., Kiecana, M., Sawicki, M., Kłopotowski, Ł., Pacuski, W. & Kossut, J. Magneto-optical properties of the diluted magnetic semiconductor *p*-type ZnMnO. *Solid State Commun.* **139**, 541–544 (2006).

[81] Pacuski, W., Suffczyński, J., Osewski, P., Kossacki, P., Golnik, A., Gaj, J. A., Deparis, C., Morhain, C., Chikoidze, E., Dumont, Y., Ferrand, D., Cibert, J. & Dietl, T. Influence of *s,p-d* and *s-p* exchange couplings on exciton splitting in $Zn_{1-x}Mn_xO$. *Phys. Rev. B* **84**, 035214 (2011).

[82] Ando, K., Saito, H., Zayets, V. & Debnath, M. C. Optical properties and functions of dilute magnetic semiconductors. *J. Phys. Cond. Matter.* **16**, S5541–S5548 (2004).

[83] Ando, K., Saito, H., Jin, Z., Fukumura, T., Kawasaki, M., Matsumoto, Y. & Koinuma, H. Magneto-optical properties of ZnO-based diluted magnetic semiconductors. *J. Appl. Phys.* **89**, 7284–7286 (2001).

[84] Mac, W., Twardowski, A. & Demianiuk, M. s,p-d exchange interaction in Cr-based diluted magnetic semiconductors. *Phys. Rev. B* **54**, 5528–5535 (1996).

[85] Twardowski, A., Swiderski, P., von Ortenberg, M. & Pauthenet, R. Magnetization and exchange constants in $Zn_{1-x}Mn_xSe$. *Solid State Commun.* **51**, 849–852 (1984).

[86] Twardowski, A., Pakula, K., Perez, I., Wise, P. & Crow, J. E. Magnetoreflectance and magnetization of the semimagnetic semiconductor $Cd_{1-x}Fe_xSe$. *Phys. Rev. B* **42**, 7567 (1990).

[87] Liu, X., Petrou, A., Jonker, B. T., Krebs, J. J., Prinz, G. A. & Warnock, J. Magnetoreflectivity study of excitons in ZnCoSe. *J. Appl. Phys.* **67**, 4796–4797 (1990).

[88] Twardowski, A., Swiderski, P., von Ortenberg, M. & Pauthenet, R. Magnetoabsorption and magnetization of $Zn_{1-x}Mn_xTe$ mixed crystals. *Solid State Commun.* **50**, 509–513 (1984).

[89] Testelin, C., Prost, J. B., Menant, M., Zielinski, M. & Mycielski, A. Magnetization and exchange interactions in $Zn_{1-x}Fe_xTe$ diluted magnetic semiconductors. *Solid State Commun.* **113**, 695–698 (2000).

[90] Zielinski, M., Rigaux, C., Lemaître, A., Mycielski, A. & Deportes, J. Exchange interactions and magnetism of Co^{2+} in $Zn_{1-x}Co_xTe$. *Phys. Rev. B* **53**, 674–685 (1996).

[91] Mac, W., Herbich, M., Twardowski, A. & Demianiuk, M. The ferromagnetic *p-d* exchange interaction in $Cd_{1-x}V_xS$. *Semicond. Sci. Technol.* **15**, 748–751 (2000).

[92] Herbich, M., Mac, W., Twardowski, A., Ando, K., Shapira, Y. & Demianiuk, M. Magnetization and exciton spectroscopy of the diluted magnetic semiconductor $Cd_{1-x}Cr_xS$. *Phys. Rev. B* **58**, 1912 (1998).

[93] Nawrocki, M., Lascaray, J. P., Coquillat, D. & Demianiuk, M. Ion-carrier exchange interaction in CdMnS. *in Diluted Magnetic (Semimagnetic) Semiconductors, edited by S. von Molnar, R. Aggarwal and J. K. Furdyna, Mat. Res. Soc. Symp. Proc.* **89**, 65 (1987).

[94] Radovanovic, P. R. & Gamelin, D. R. Magnetic circular dichroism spectroscopy of Co^{2+}:CdS diluted magnetic semiconductor quantum dots. *Proc. SPIE* **4809**, 51–61 (2002).

[95] Arciszewska, M. & Nawrocki, M. Determination of the band structure parameters of $Cd_{0.95}Mn_{0.05}Se$ from magnetoabsorption measurements. *J. Phys. Chem. Solids* **47**, 309–314 (1986).

[96] Scalbert, D., Guillot, M., Mauger, A., Gaj, J. A., Cernogora, J., Benoit à la Guillaume, C. & Mycielski, A. High field magnetization and exchange integrals in $Cd_{1-x}Fe_xSe$. *Solid State Commun.* **76**, 977–980 (1990).

[97] Nawrocki, M., Hamdani, F., Lascaray, J. P., Golacki, Z. & Deportes, J. Ion-carrier electron exchange constants for CdCoSe semimagnetic semiconductor. *Solid State Commun.* **77**, 111–114 (1991).

[98] Gaj, J., Planel, R. & Fishman, G. Relation of magnetooptical properties of free excitons to spin alignment of Mn^{++} ions in $Cd_{1-x}Mn_xTe$. *Solid State Commun.* **29**, 435–438 (1979).

[99] Testelin, C., Rigaux, C., Mycielski, A., Menant, M. & Guillot, M. Exchange interactions in CdFeTe semimagnetic semiconductors. *Solid State Commun.* **78**, 659–663 (1991).

[100] Zielinski, M., Rigaux, C., Mycielski, A. & Menant, M. Zeeman spectrum of the 1s exciton in very diluted $Cd_{1-x}Co_xTe$ compounds. *Phys. Rev. B* **63**, 035202 (2000).

[101] Alawadhi, H., Miotkowski, I., Souw, V., McElfresh, M., Ramdas, A. K. & Miotkowska, S. Excitonic Zeeman effect in the zinc-blende II-VI diluted magnetic semiconductors $Cd_{1-x}Y_xTe$ (Y=Mn, Co, and Fe). *Phys. Rev. B* **63**, 155201 (2001).

[102] Pacuski, W., Ferrand, D., Cibert, J., Gaj, J. A., Golnik, A., Kossacki, P., Marcet, S., Sarigiannidou, E. & Mariette, H. Excitonic giant zeeman effect in GaN:Mn^{3+}. *Phys. Rev. B* **76**, 165304 (2007).

[103] Suffczyński, J., Grois, A., Pacuski, W., Golnik, A., Gaj, J. A., Navarro-Quezada, A., Faina, B., Devillers, T. & Bonanni, A. Effects of *s*,*p*-*d* and *s*-*p* exchange interactions probed by exciton magnetospectroscopy in (Ga,Mn)N. *Phys. Rev. B* **83**, 094421 (2011).

[104] Pacuski, W., Kossacki, P., Ferrand, D., Golnik, A., Cibert, J., Wegscheider, M., Navarro-Quezada, A., Bonanni, A., Kiecana, M., Sawicki, M. & Dietl, T. Observation of strong-coupling effects in a diluted magnetic semiconductor $Ga_{1-x}Fe_xN$. *Phys. Rev. Lett.* **100**, 037204 (2008).

[105] Szczytko, J., Mac, W., Twardowski, A., Matsukura, F. & Ohno, H. Antiferromagnetic *p*-*d* exchange in ferromagnetic $Ga_{1-x}Mn_xAs$ epilayers. *Phys. Rev. B* **59**, 12935–12939 (1999).

[106] Léger, Y., Besombes, L., Maingault, L., Ferrand, D. & Mariette, H. Geometrical effects on the optical properties of quantum dots doped with a single magnetic atom. *Phys. Rev. Lett.* **95**, 047403 (2005).

[107] Maingault, L., Besombes, L., Léger, Y., Bougerol, C. & Mariette, H. Inserting one single Mn ion into a quantum dot. *Appl. Phys. Lett.* **89**, 193109 (2006).

[108] Li, X., Yang, W., Chang, K. & Xia, J. Spin states in semiconductor quantum dot with a single magnetic ion. *Physica E* **40**, 3097–3106 (2008).

[109] Le Gall, C., Brunetti, A., Boukari, H. & Besombes, L. Optical stark effect and dressed exciton states in a Mn-doped CdTe quantum dot. *Phys. Rev. Lett.* **107**, 057401 (2011).

[110] Le Gall, C., Kolodka, R. S., Cao, C. L., Boukari, H., Mariette, H., Fernández-Rossier, J. & Besombes, L. Optical initialization, readout, and dynamics of a Mn spin in a quantum dot. *Phys. Rev. B* **81**, 245315 (2010).

[111] Gietka, K., Kobak, J., Rousset, J.-G., Janik, E., Supiski, T., Kossacki, P., Golnik, A. & Pacuski, W. MBE growth of CdTe/ZnTe quantum dots with single Mn ions. *Acta Phys. Pol. A* **122**, 1056–1058 (2012).

[112] Mendes, U. C., Korkusinski, M., Trojnar, A. H. & Hawrylak, P. Optical properties of charged quantum dots doped with a single magnetic impurity. *Phys. Rev. B* **88**, 115306 (2013).

[113] Thuberg, D., Reiter, D. E., Axt, V. M. & Kuhn, T. Switching between ground states of an InAs quantum dot doped with a single Mn atom. *Phys. Rev. B* **88**, 085312 (2013).

[114] Kulakovskii, V. D., Bacher, G., Weigand, R., Kümmell, T., Forchel, A., Borovitskaya, E., Leonardi, K. & Hommel, D. Fine structure of biexciton emission in symmetric and asymmetric CdSe/ZnSe single quantum dots. *Phys. Rev. Lett.* **82**, 1780–1783 (1999).

[115] Patton, B., Langbein, W. & Woggon, U. Trion, biexciton, and exciton dynamics in single self-assembled CdSe quantum dots. *Phys. Rev. B* **68**, 125316 (2003).

[116] Suffczyński, J., Kazimierczuk, T., Goryca, M., Piechal, B., Trajnerowicz, A., Kowalik, K., Kossacki, P., Golnik, A., Korona, K. P., Nawrocki, M., Gaj, J. A. & Karczewski, G. Excitation mechanisms of individual CdTe/ZnTe quantum dots studied by photon correlation spectroscopy. *Phys. Rev. B* **74**, 085319 (2006).
